# Light‐driven synthetic microbial consortia: playing with an oxygen dilemma

**DOI:** 10.15302/J-QB-022-0314

**Published:** 2023-06-01

**Authors:** Huawei Zhu, Yin Li

**Affiliations:** ^1^ CAS Key Laboratory of Microbial Physiological and Metabolic Engineering State Key Laboratory of Microbial Resources Institute of Microbiology Chinese Academy of Sciences Beijing 100101 China

**Keywords:** synthetic microbial consortia, oxygen dilemma, photosynthesis

## Abstract

**Background:**

Light‐driven synthetic microbial consortia are composed of photoautotrophs and heterotrophs. They exhibited better performance in stability, robustness and capacity for handling complex tasks when comparing with axenic cultures. Different from general microbial consortia, the intrinsic property of photosynthetic oxygen evolution in light‐driven synthetic microbial consortia is an important factor affecting the functions of the consortia.

**Results:**

In light‐driven microbial consortia, the oxygen liberated by photoautotrophs will result in an aerobic environment, which exerts dual effects on different species and processes. On one hand, oxygen is favorable to the synthetic microbial consortia when they are used for wastewater treatment and aerobic chemical production, in which biomass accumulation and oxidized product formation will benefit from the high energy yield of aerobic respiration. On the other hand, the oxygen is harmful to the synthetic microbial consortia when they were used for anaerobic processes including biohydrogen production and bioelectricity generation, in which the presence of oxygen will deactivate some biological components and compete for electrons.

**Conclusions:**

Developing anaerobic processes in using light‐driven synthetic microbial consortia represents a cost‐effective alternative for production of chemicals from carbon dioxide and light. Thus, exploring a versatile approach addressing the oxygen dilemma is essential to enable light‐driven synthetic microbial consortia to get closer to practical applications.

## Abstract 2

**Author summary:** Oxygen plays a significant role affecting the functions of synthetic microbial consortia. In a light‐driven synthetic microbial consortium, the compacted space makes heterotrophs easily access to the oxygen produced by photoautotrophs. Thus, the sensitivity and requirement of oxygen for heterotrophs involved in a light‐driven microbial consortium determines the overall performance of the process. Once an obligate anaerobe or an anaerobic metabolic process was introduced, the oxygen must be isolated from heterotrophs to avoid oxygen inactivation and oxygen respiration. This article discussed how to play with the oxygen dilemma in light‐driven synthetic microbial consortia to maximize the performance of the systems.

## INTRODUCTION

Synthetic microbial consortia have received increasing attentions in synthetic biology, synthetic ecology and biotechnology fields. Compared with axenic cultures using single microbes, synthetic microbial consortia exhibited easiness in modular engineering, expanded metabolic capabilities, reduced metabolic burden, stronger robustness and increased efficiency [1, 2, 3]. The biotechnological potentials of using synthetic microbial consortia for productions of biofuels, chemicals and biohydrogen, as well as for environmental bioremediation and bioelectricity generation, have been well documented [4, 5, 6, 7].

Light‐driven synthetic microbial consortia are defined as the synthetic microbial consortia composed of photoautotrophs and heterotrophs. They are capable of directly converting carbon dioxide (CO_2_) into fuels, materials and chemicals using the energy stored in the light. Generally, the photosynthetic microorganisms in a light‐driven synthetic microbial consortium absorb light and fix CO_2_ into organic carbon to support the growth of heterotrophic microorganisms. In turn, the CO_2_ released by heterotrophic partners can be taken up by its photosynthetic partners [8]. In this connection, light‐driven synthetic microbial consortia can be considered carbon‐negative in terms of the sum of the consumption and release of CO_2_. Thus, light‐driven synthetic microbial consortia seem to be a good approach to contribute to carbon neutrality. However, the efficiency of the light‐driven synthetic microbial consortia is often affected by the oxygen generated by photoautotrophs. The oxygen produced by photoautotrophs may facilitate the growth of heterotrophs and organics oxidation if the heterotrophs are aerophilic. However, in those processes where oxygen is deleterious to the growth and metabolism of heterotrophs, the oxygen released by photoautotrophs becomes a detrimental factor that needs to be removed or attenuated.

**Figure 1:**
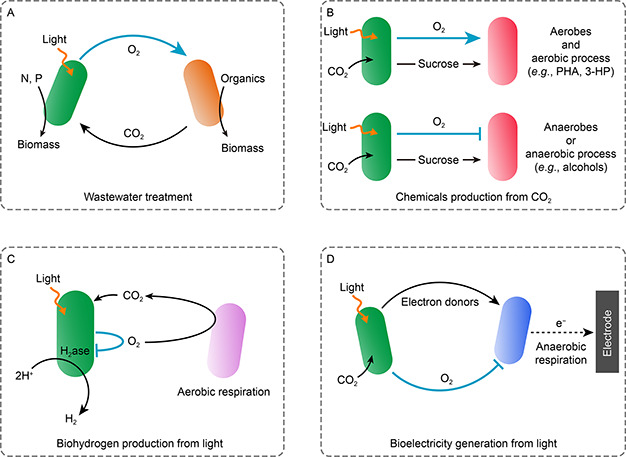
**The oxygen dilemma in light‐driven synthetic microbial consortia.** (A) Oxygen favors light‐driven microbial consortia for wastewater treatment. The cross‐feeding of O_2_ and CO_2_ between photosynthetic partner and heterotrophic partner is favorable to them for simultaneous removal of organic and inorganic pollutants from wastewater. (B) In the light‐driven synthetic microbial consortia used for chemical production, oxygen is beneficial to the aerobic processes of aerobes, such as the production of PHA and 3‐HP. However, oxygen is detrimental to the anaerobes or the anaerobic processes of facultative anaerobes, such as the production of alcohols. (C) The hydrogenases (H_2_ase) responsible for biohydrogen production are extremely sensitive to oxygen, which could be eliminated by heterotrophic partners through aerobic respiration. (D) In the lightdriven synthetic microbial consortia used for bioelectricity generation, the presence of oxygen will compete with extracellular respiration for electrons, leading to low electron extraction efficiency. The aerophilic effects and aerophobic effects were indicated with blue arrows and blue blunt arrows, respectively.

This review summarized recent advances in the applications of light‐driven synthetic microbial consortia, with focus on addressing the oxygen dilemma illustrated above. We categorized these applications as aerophilic processes such as wastewater treatment and chemicals production, and aerophobic processes such as biohydrogen production and bioelectricity generation ( Fig.1). Moreover, the strategies to address the oxygen dilemma were discussed and future perspectives for developing more efficient light‐driven synthetic microbial consortia were proposed.

## OXYGEN FAVORS LIGHT‐DRIVEN MICROBIAL CONSORTIA FOR WASTEWATER TREATMENT

Municipal and industrial wastewaters contain organic carbon and inorganic nutrients such as nitrogen and phosphorous, which may result in water eutrophication if not treated properly. Microalgae assimilates nitrogen and phosphorus into biomass, which can subsequently be used as fertilizer or renewable feedstock for biofuels production [9]. Numerous algae strains have been used in wastewater treatment processes [10]. However, the efficiency of nitrogen and phosphorous removal by single microalgae is low. In addition, microalgae cannot remove the organic pollutants present in wastewaters. By combining the oxidation ability of bacteria, microalgae‐bacteria consortia showed greater potential to achieve high efficiency in simultaneous removal of organic and inorganic pollutants from wastewater [9].

The increased efficiency of treating wastewater by microalgae‐bacteria consortia is benefited from the symbiotic relationship between these microorganisms. Under light conditions, microalgae perform photosynthesis to assimilate CO_2_, nitrogen and phosphorous nutrients into biomass, and meanwhile producing oxygen. The released oxygen is used by aerobic heterotrophs to perform aerobic respiration for the degradation of organic carbon or nitrification for the elimination of ammonium [11,12]. Concurrently, heterotrophs release CO_2_ which is subsequently taken up again by microalgae. In such a microalgae‐bacteria consortium, the oxygen is a prerequisite for degradation of organic matters, and the decreased dissolved oxygen concentration in the culture due to constant consumption by heterotrophs is also important for maintaining the photosynthesis rate of microalgae [13]. In this consortium, molecular oxygen plays a positive role for facilitating removal of pollutants.

The degradation of organic pollutants from wastewater by activated sludge is an aerobic process. Supplying oxygen through mechanical aeration is an energy‐intensive process, which accounts for more than 50% of the treatment cost [9,14,15]. Thus, using oxygen generated from photosynthesis by microalgae‐bacteria consortia is an economic alternative to replace mechanical aeration in wastewater treatment ( Tab.1). For instance, a co‐culture of microalgae *Chlorella vulgaris* and *Pseudomonas putida* achieved a complete organic carbon removal, compared to that of 50% without photosynthetic microalgae [15]. Moreover, the co‐culture of *C. vulgaris* and *P. putida* could simultaneously remove inorganic nutrients and organic carbon with efficiencies higher than that of the corresponding axenic cultures [17,19]. The removal efficiencies of nitrogen, phosphorus, and organic carbon by co‐culture system were 85%, 66% and 86%, respectively, in contrast to those of 13%, 0% and 72% for pure culture of *P. putida* [19]. Using co‐culture of *Scenedesmus dimorphus* and nitrifiers, an improvement of 3.4‐fold for total nitrogen (TN) removal and 6.5‐fold for total phosphate (TP) removal were achieved, respectively, compared to that of the nitrifiers‐only systems [20]. Similarly, a higher removal efficiency of TN (86%) and TP (93%) were achieved in a co‐culture of *C. vulgaris* and *Bacillus licheniformis*, compared to that of the system containing either single algae or single bacteria [18]. Besides the superior wastewater treatment performance, the co‐culture composed of *C. vulgaris* and *Rhizobium* sp. also led to a 3‐fold higher biomass accumulation with a 13‐fold higher fatty acid content compared to that of the axenic algal culture [16]. In these co‐culture treatment systems, sufficient oxygen was provided via microalgae photosynthesis, thereby satisfying the oxygen requirement for heterotrophs.

The growth of heterotrophs is benefited from the oxygen generated from photosynthesis, while on the other hand oxygen consumption also increased algal growth. Previous studies reported that high oxygen levels could inhibit the photosynthesis of some microalgae [11]. Once the dissolved oxygen concentration reached 20−30 mg L^−1^, the photosynthesis rate would diminish to zero [11]. This is because the dissolved oxygen can lower the net photosynthetic carbon fixation by favoring the oxygenase activity of the ribulose‐1,5‐bisphosphate carboxylase/oxygenase (Rubisco) [9]. Under normal growth conditions, the photosynthetic rate of microalgae is usually 4−7 folds higher than its respiration rate, thereby resulting oxygen inhibition [9]. Hence, reducing oxygen level by heterotrophic partner is one of the reasons accounting for algal growth enhancement in microalgae‐bacteria consortia. As a consequence, the oxygen is favorable for a light‐driven microbial consortium to treat wastewater efficiently.

**Table 1:** The light‐driven synthetic microbial consortia for wastewater treatment

Photoautotrophs	Heterotrophs	Wastewater sources	Performance		The effects of oxygen		References
*Chlorella vulgaris*	*Rhizobium* sp.	Synthetic municipal wastewater	3‐fold higher biomass, 13‐fold higher fatty acid, 41%~58% higher TOC/TN/TP removal efficiency relative to axenic algal culture		Microalgae‐generated oxygen supported the growth of *Rhizobium*		[16]
*Chlorella vulgaris*	*Pseudomonas putida*	Synthetic municipal wastewater	Better performance in both nutrients and COD removal (around 80% removal) than each of axenic cultures		Microalgae‐generated oxygen served as an electron acceptor of *P. putida*		[17]
*Chlorella vulgaris*	*Pseudomonas putida*	Synthetic municipal wastewater	Removal efficiency of organics improved from 50% without aeration to 100% when photosynthetic aeration conducted		Microalgae‐generated oxygen achieved *in situ* photosynthetic aeration for organic removal by *P. putida*		[15]
*Chlorella vulgaris*	*Bacillus licheniformis*	Modified OECD medium	Higher removal efficiencies of NH_4_^+^ (86%) and TP (93%) than single algae system or single bacteria system		Microalgae‐generated oxygen supported the growth of *B. licheniformis*, and the latter in turn promoted the growth of *C. vulgaris*		[18]
*Chlorella vulgaris*	*Pseudomonas putida*	Synthetic municipal wastewater	Higher removal of both nutrients and COD than the each axenic culture		*P. putida* mineralize organic carbons by consuming the dissolved oxygen in wastewater		[19]
*Scenedesmus dimorphus*	Nitrifiers (enriched activated sludge)	Artificial wastewater	3.4‐fold higher TN removal efficiency and 6.5‐fold higher TP removal efficiency compared to nitrifiers‐only reactors		Microalgae maintained high dissolved oxygen without external aeration		[20]

Abbreviations: TOC: total organic carbon; TN: total nitrogen; TP: total phosphate; COD: chemical oxygen demand.

## OXYGEN FAVORS LIGHT‐DRIVEN MICROBIAL CONSORTIA FOR AEROBIC PRODUCTION OF CHEMICALS

Light‐driven microbial consortia offer a promising alternative of converting CO_2_ into various chemicals using the energy from light. Metabolic engineering enables cyanobacteria to produce extracellular sucrose from light and CO_2_ under osmotic pressure environment [21]. Sucrose synthesis in cyanobacteria showed a higher production rate compared to most of the photosynthetic products and its carbon partitioning was close to 85% [22]. Photosynthesis‐derived sucrose provides a carbohydrate source for the well‐established heterotrophic microbial factories such as *Escherichia coli* and *Saccharomyces cerevisiae* to produce valuable chemicals [23]. To date, the synthesized end‐products by light‐driven synthetic microbial consortia mainly include polyhydroxyalkanoates (PHA), 3‐hydroxypropionic acid (3‐HP) and fatty acids ( Tab.2).

Ducat *et al.* [32] firstly proved that the sucrose secreted by an engineered cyanobacterium *Synechococcus elongatus* PCC 7942 could support the growth of *S. cerevisiae*. Further study showed that the growth of *S. elongatus* could also be enhanced by heterotrophs including *E. coli*, *S. cerevisiae*, and *Bacillus subtilis* [33]. Meanwhile, the desired chemicals such as polyhydroxybutyrate (PHB) and α‐amylase could be produced when engineered *E. coli* and *B. subtilis* was respectively incorporated into the light‐driven synthetic microbial consortia [33]. An improved version of light‐driven PHB‐synthesizing microbial consortium was constructed by using a natural PHB producer *Halomonas boliviensis* and a sucrose‐secreting *S. elongatus* encapsulated in alginate hydrogel [24]. Alginate encapsulation of cyanobacteria restricted its growth while increased the rate of sucrose production. Meanwhile, encapsulation could facilitate the separation and collection of *H. boliviensis* from co‐cultures, thereby enabling long‐term production and stability. Finally, this synthetic microbial consortium was able to synthesize PHB continuously for over 5 months and reached a productivity of 28.3 mg L^−1^ d^−1^, exceeding those of the engineered cyanobacteria monocultures [24]. Moreover, Smith *et al.* [25] chose a diazotroph *Azotobacter vinelandii* as a heterotrophic partner, forming a cross‐feeding microbial consortium where the fixed carbon and the fixed nitrogen were shared. This design enabled biomass accumulation and PHB production solely from CO_2_ and light in the absence of nitrogen sources [25]. Although PHB production took place under aerobic conditions, the optimal PHB accumulation occurred when oxygen was limiting [25,34]. Oxygen limitation might inhibit TCA cycle, which is the most important competing pathway for PHB production. To acquire an oxygen‐limiting condition in the light‐driven synthetic microbial consortium, some strategies, such as aeration of inert gases or cultivation of PHB‐producer underneath the photosynthetic partners, are necessary to be taken.

**Table 2:** The light‐driven synthetic microbial consortia for chemical production

Photoautotrophs	Heterotrophs	Chemicals	Performance		Comments on oxygen		References
*Synechococcus elongatus* PCC 7942	*Halomonas boliviensis*	PHB	28.3 mg L^−1^ d^−1^ of PHB productivity, 5 months of continuous production		PHB production can undertake in aerobic conditions but optimal PHB accumulation occurs when oxygen is limiting		[24]
*Synechococcus elongatus* PCC 7942	*Azotobacter vinelandii*	PHB	PHB content accounted for 20% dry cell weight				[25]
*Synechococcus elongatus* PCC 7942	*Pseudomonas putida* (*cscAB*)	PHA	23.8 mg L^−1^ d^−1^ of PHA productivity, 156 mg L^−1^ of maximal PHA titer		PHA production is often an aerobic process, while the operating expense for aeration is high. PHA producer will benefited from photosynthetic oxygen evolution when co‐cultured with cyanobacteria		[26]
*Synechococcus elongatus* PCC 7942	*Pseudomonas putida* (EM∙DNT∙S)	PHA	Simultaneous removal of 2,4‐dinitrotoluene and PHA accumulation PHA content of 23.4 mg g^−1^ dry cell weight				[27]
*Synechococcus elongatus* PCC 7942	*Pseudomonas putida* (*cscRABY ∆nasT*)	PHA	42.1 mg L^−1^ d^−1^ of PHA productivity, 393 mg L^−1^ of maximal PHA titer				[28]
*Synechococcus elongatus* UTEX 2973	*Escherichia coli* (ABKm)	3‐HP	Producing 3‐HP at up to 68.29 mg L^−1^ directly from CO_2_		The growth enhancement of *S. elongatus* was probably due to the quenching of ROS by *E. coli*		[29]
*Synechococcus elongatus* PCC 7942	*Rhodotorula glutinis*	Fatty acids	The total biomass and lipid yield in co‐culture were 40% to 60% higher than the mono‐culture of *S. elongatus*		The ROS‐induced growth inhibition of *S. elongatus* could be alleviated by *R. glutinis*		[30]
*Nostoc* PCC 7413	*Aspergillus nidulans*	Fatty acids	The total biomass was 3‐fold higher than the mono‐culture of *Nostoc*		Aerobic fungi *A. nidulans* could benefit from oxygen‐producing cyanobacteria		[31]

Microbial PHA is produced under aerobic conditions, while the operating expense for aeration is high [35]. Once co‐cultured with cyanobacteria, the microbial PHA producer will be benefited from the oxygen generated from photosynthesis. The microbial consortia containing sucrose‐secreting *S. elongatus* and PHA producer *P. putida* were constructed for PHA production [26, 27, 28]. The highest PHA productivity from light‐driven microbial consortia reported to date was 42.1 mg L^−1^ d^−1^, where an engineered *P. putida* capable of efficiently utilizing sucrose was used [28]. In addition, another engineered *P. putida* capable of degrading 2,4‐dinitrotoluene was used in microbial consortia, allowing simultaneous removal of 2,4‐dinitrotoluene and accumulation of PHA from CO_2_ and light [27]. In this consortium, the oxygen derived from photosynthesis was also in favor of the aerobic degradation of 2,4‐dinitrotoluene [27]. In the aforementioned light‐driven microbial consortia developed for chemical production, the sucrose production chassis is *S. elongatus* PCC 7942. In contrast, sucrose production of a fast‐growing cyanobacterium *S. elongatus* UTEX 2973 was 2‐fold faster than that of *S. elongatus* PCC 7942 [36,37]. Using the co‐culture of *S. elongatus* UTEX 2973 and an engineered *E. coli*, an important platform chemical 3‐HP could be produced at 68.29 mg L^−1^ [29]. This demonstrated the feasibility of converting CO_2_ into 3‐HP using light‐driven synthetic microbial consortium, although the productivity was approximately 10‐fold lower than that of axenic culture of the engineered cyanobacteria [38]. The researchers further proved that *E. coli* could promote the growth of *S. elongatus* UTEX 2973 by quenching reactive oxygen species (ROS) produced by cyanobacteria [29,39].

The mutual growth enhancement in light‐driven microbial consortia could be used for fatty acids production. Oleaginous yeasts such as *Cryptococcus curvatus* and *Rhodotorula glutinis* could accumulate approximately 70% lipids in their biomass [30], thus serving as a heterotrophic partners in synthetic microbial consortia for fatty acids production from CO_2_ and light. Notably, dissolved oxygen level is one of the limiting factors affecting the biomass accumulation of these aerobic oleaginous yeasts [8]. In a co‐culture composed of sucrose‐secreting *S. elongatus* and lipid‐producing *R. glutinis*, the total biomass and total fatty acids content increased by 40%−60% compared with mono‐cultures of *S. elongatus* and *R. glutinis* [30]. Besides sucrose, the extracellular polysaccharide (EPS) secreted by cyanobacterium *Nostoc* PCC 7413 could support the growth of *Aspergillus nidulans* [31]. The total biomass in this co‐culture was approximately 3‐fold higher than that of the axenic *Nostoc* culture, resulting in higher fatty acids accumulation. The other chemicals produced by light‐driven synthetic microbial consortia include furandicarboxylic acid [40], isoprene [41] and astaxanthin [42].

As described above, the target chemicals produced by light‐driven synthetic microbial consortia mainly included aerobic fermentation chemicals and biomass‐derived chemicals. Both kinds of chemicals are benefited from *in situ* oxygen supply by photosynthetic microorganisms. The increased dissolved oxygen during photosynthesis promoted the growth of heterotrophic partners, and the consumption of oxygen by heterotrophic microorganisms eliminated the hyperoxia inhibition on photosynthesis rate. However, the product yield in the presence of oxygen is generally low because part of the organic matter generated by photoautotrophs was completely oxidized into CO_2_. In other words, lower yields and productivities are inherent to the presence of aerobic respiration [43]. To date, the highest titer obtained in light‐driven microbial consortia was 393 mg L^−1^, with a carbon yield of lower than 20% [28]. High carbon yield for production of chemicals is often associated with anaerobic conditions, but developing an anaerobic process in the light‐driven microbial consortia is challenging. In addition, the low productivity of sucrose is certainly a limit for the industrial application of light‐driven synthetic microbial consortia. For that, more systematical engineering of cyanobacteria chassis is needed.

## OXYGEN IS DETRIMENTAL TO LIGHT‐DRIVEN MICROBIAL CONSORTIA FOR HYDROGEN PRODUCTION

Molecular hydrogen (H_2_) is a clean and high energy‐density alternative to fossil fuel. Hydrogen could be biologically produced by green algae, cyanobacteria, anoxygenic photosynthetic bacteria and some heterotrophic bacteria through photosynthesis or fermentation [44]. Among them, biological hydrogen production by green algae and cyanobacteria has received considerable attention since they do not require organic carbon sources [45]. The hydrogen production in green algae is predominantly contributed by [FeFe] hydrogenase, and its activity is 10 to 100‐fold higher than [NiFe] hydrogenase in cyanobacteria [46]. However, [FeFe] hydrogenase is highly sensitive to oxygen, which was documented to be the main bottleneck for algal photolysis H_2_ production. Some solutions have been implemented to lower the oxygen level of algal culture, such as sulfur deprivation for partially inactivating PSII activity, the addition of oxygen scavengers, and removal oxygen using inert gas [47,48].

Co‐cultivation of microalgae with aerobic bacteria is another approach to create hypoxia atmosphere for enabling the hydrogenases to work. Many studies have demonstrated the possibility of increasing H_2_ production by co‐culturing microalgae and bacteria. A recent review provided a comparative analysis of previously published data about hydrogen production in *Chlamydomonas*‐bacteria consortia with their respective algal monocultures [49]. It was shown that the hydrogen productivity in many *Chlamydomonas*‐bacteria exhibited at least three‐fold enhancements relative to the algal mono‐cultures. Using co‐cultures with *P. putida* or *Bradyrhizobium japonicum* as oxygen‐consuming partners, the H_2_ production of *C. reinhardtii* improved 23 times and 32 times compared to that of the algal mono‐cultures, respectively [49, 50, 51]. Notably, these two bacterial partners are not known to produce H_2_ by themselves. Besides pure culture of bacteria, the aerobic microbial community present in activated sludge could efficiently scavenge oxygen, which represents a natural oxygen‐consuming partner without requirement of maintaining the purity of bacteria [52]. Moreover, the fast oxygen depletion by aerobic bacteria also enable hydrogen production by light‐driven microbial consortia under high light intensity (higher than 100 PPFD, *i.e.*, μmol photons m^−2^ s^−1^), whereas the optimal light intensity used for hydrogen generation of pure *C. reinhardtii* is generally below 50 PPFD [49,53]. For example, the maximum hydrogen yields of pure *C. reinhardtii* under light intensities of 60, 100, 200 PPFD were 41, 23 and 16 μmol mg^−1^ Chl, respectively, which means high light intensity inhibited hydrogen production [54]. In contrast, the maximum hydrogen yields of *C. reinhardtii*‐ *B. japonicum* co‐culture under above light intensities were 60, 175 and 272 μmol mg^−1^ Chl, respectively [54]. This indicated light‐driven microbial consortia could produce hydrogen under high light intensity and the corresponding hydrogen yields were much higher than those of mono‐cultures. In another study, the optimal hydrogen production for pure *C. reinhardtii* occurred at 12 PPFD, while almost no H_2_ was produced under 100 PPFD [51]. However, the optimal hydrogen production for the co‐culture with *Pseudomonas* sp. was obtained at a high light intensity of 100 PPFD [51]. Similarly, when light intensity increased from 30 up to 200 PPFD, the hydrogen production of pure *C. reinhardtii* was almost stopped, while the hydrogen production rate of *C. reinhardtii*‐ *B. japonicum* co‐culture increased by 3‐fold [50]. Taken together, although oxygen accumulated in the mono‐culture of algae is deleterious to the process of photosynthesis hydrogen production, the light‐driven microbial consortia could well address the oxygen issue, providing a promising route for biohydrogen production.

## OXYGEN IS DETRIMENTAL TO LIGHT‐DRIVEN MICROBIAL CONSORTIA FOR BIOELECTRICITY GENERATION

Bioelectricity represents a green and renewable energy. It can be generated in microbial fuel cells (MFCs) by heterotrophic electroactive bacteria or biophotovoltaic systems (BPVs) by photosynthetic microorganisms [55,56]. However, the bioelectricity generation in MFCs is dependent on the supply of exogenous organic substrates, whereas the power outputs of BPVs were extremely low due to the weak exoelectrogenic activity of photosynthetic microorganisms [57]. Recently, some studies reported that bioelectricity generation could also be accomplished by the light‐driven microbial consortia that composed of photosynthetic microorganisms and electroactive bacteria ( Tab.3). In such a microbial consortium, photosynthetic microorganisms use light to produce organic matter, which is subsequently converted into electricity by electroactive bacteria [65]. Thus, the light‐driven microbial consortia are theoretically superior to MFCs and BPVs in terms of sustainability and power output, respectively.

The light‐driven bioelectricity generation was firstly demonstrated in natural microbial communities [58,59,66, 67, 68, 69]. A sediment‐type phototrophic MFC was developed for continuously generating electricity without the input of exogenous organics or nutrients [69]. In this system, the photosynthetic microorganisms and electroactive bacteria were enriched from the inoculum that contained lake water and sediment. A negative light response of electrical current under light‐dark periods was observed, which was possibly due to the presence of oxygen produced by photosynthetic microorganisms during the light periods [69]. The light‐driven bioelectricity‐generating systems were further developed using defined photosynthetic microorganisms. Zhang *et al.* [59] constructed a microbial consortium consisting of microalgae *C. vulgaris* and electroactive bacteria enriched from sediments. Under illumination, this system generated a stable power density of 68 mW m^−2^ and removed carbon, nitrogen and phosphorus from wastewater simultaneously. In another study, a microbial consortium was constructed by coupling microalgae *C. vulgaris* and an electroactive culture enriched from activated sludge [58]. This system generated electrical energy without an external supply of organic substrate, which was provided by microalgae. In both systems, the inhibitory effects of oxygen accumulation on current generation were observed [58,59].

**Table 3:** The light‐driven synthetic microbial consortia for bioelectricity generation

Photoautotrophs	Electroactive bacteria(heterotrophs)	Energy carriers	Power densities (mW m^−2^)	Effects of oxygen	References
*Chlorella vulgaris*	Activated sludge sample	Undefined	−	Negative	[58]
*Chlorella vulgaris*	Sediment sample	Undefined	68	Negative	[59]
*Chlamydomonas reinhardtii*	*Geobacter sulfurreducens*	Formate	41	Negative	[60]
*Synechocystis* sp. PCC 6803	*Shewanella oneidensis* MR‐1	Undefined	−	Negative	[61]
*Synechocystis* sp. PCC 6803	*Shewanella oneidensis* MR‐1,*Pseudomonas aeruginosa* PA01	Undefined	600	Negative	[62]
*Synechococcus elongatus* UTEX 2973 (engineered)	*Shewanella oneidensis* MR‐1(engineered)	d‐lactate	150	Negative	[63]
*Synechococcus elongatus* PCC 7942 (engineered)	*Escherichia coli* (engineered), *Shewanella oneidensis* MR‐1 (engineered), *Geobacter sulfurreducens*	Sucrose	1700	Negative	[64]

To better understand the energy‐conversion mechanisms in light‐driven microbial communities, it is important to use synthetic microbial consortium with well‐defined composition of microbial species. The genera *Geobacter* and *Shewanella* are two well‐studied electroactive bacteria capable of converting organic carbon into electricity [70], and thereby suitable to be used in the defined microbial consortia. Nishio *et al.* [60] constructed a defined co‐culture of *C. reinhardtii* and *G. sulfurreducens* for light‐electricity conversion. It was demonstrated that the formate produced by *C. reinhardtii* was the major electron donor of *G. sulfurreducens*, and they also found that the oxygen accumulated under illumination might lead to the decrease of current during the light periods [60]. Additionally, Liu *et al*. [61] created a light‐driven MFC using a photosynthetic‐heterotrophic microbial consortium consisting of *Synechocystis* sp. PCC 6803 and *Shewanella oneidensis* MR‐1. This synthetic microbial consortium achieved a self‐sustaining electricity generation for 13 days without additional substrates. Similarly, a negative light response of electrical current was attributed to the negative effect of photosynthetic oxygen evolution, which diverted electrons away from the anode [61]. Moreover, Liu *et al*. [62] constructed a three‐species microbial consortium composed of *Synechocystis* sp. PCC 6803 and two electroactive bacteria. In this consortium, *S. oneidensis* MR‐1 and *Pseudomonas aeruginosa* PA01 were simultaneously introduced for electricity generation by consuming the organic matter produced by cyanobacterium, which achieved a high power output of 600 mW m^−2^ [62]. In this system, three microbial species were vertically separated from each other by using solid‐state agar to avoid the penetrating of oxygen into the electroactive bacteria [62].

In above studies, only a small amount of organic carbon was released to the extracellular medium since the wile‐type photosynthetic microorganisms were used, thereby limiting the supply of electron donors. To increase the electron donors, we developed an advanced microbial consortium composed of an engineered photosynthetic cyanobacterium and *S. oneidensis* MR‐1 [63]. The cyanobacterium *Synechococcus elongatus* UTEX 2973 was genetically engineered to synthesize d‐lactate, which is the preferential electron donor of *S. oneidensis* MR‐1. An estimated 10.3% of total fixed light energy was directed into d‐lactate used for electricity generation. In this microbial consortium, the electrons flowed from photons to d‐lactate, then to electricity, and eventually achieving a high power density of 150 mW m^−2^. Moreover, to overcome the oxygen competition for electrons, we developed a spatial‐temporal separation setup to assemble cyanobacteria‐ *Shewanella* consortium. In a spatial‐temporal separation setup with minimal inorganic nutrients replenishment, this microbial consortium achieved a steady power output for over 40 days. Recently, a four‐species microbial consortium composed of cyanobacteria, *E. coli*, *S. oneidensis* and *G. sulfurreducens* was used for electricity generation from light. In this synthetic consortium, approximately two thirds of the solar energy fixed by the cyanobacteria (in the form of sucrose) was converted into electricity, and a maximum power density of 1700 mW m^−2^ was obtained on a porous electrode [64]. In summary, the light‐driven microbial consortia have shown great potential for improving power output and prolonging the operating stability of systems. However, the contradiction between photosynthetic oxygen production and anaerobic electricity generation remains to be an obstacle that restricts the energy efficiency and increases the operation difficulty of these systems.

## PERSPECTIVES

In a light‐driven synthetic microbial consortium, the photosynthetic partner produces massive oxygen during photosynthesis. When the heterotrophic partner was an aerobic microorganism, the cross‐feeding of O_2_ and CO_2_ between photosynthetic partner and heterotrophic partner is favorable to their growth and biomass accumulation. Therefore, the liberated oxygen through photosynthesis could promote the processes of aerobic chemical production and wastewater treatment, where the removals of inorganic nutrients and organic carbon pollutants were dependent on biomass assimilation and aerobic oxidation, respectively. Moreover, the *in situ* photosynthetic aeration also saved the cost of physical aeration for these aerobic processes. However, the photosynthesis‐derived oxygen is lethal to the anaerobic microbial processes, such as biohydrogen production, bioelectricity generation and anaerobic chemical production. On one hand, when the heterotrophic partner is obligate anaerobe like *Geobacter* and *Clostridium*, the presence of oxygen will cause cell death. Additionally, the high dissolved oxygen will deactivate the oxygen‐sensitive enzymes like hydrogenases. On the other hand, the presence of oxygen will compete with other metabolic pathways for electrons, which will lead to low carbon yield for chemical production and low electron extraction efficiency for bioelectricity generation.

The physiological incompatibility of oxygen among microbial community members can be resolved via spatial and temporal separation, which allowed creating individualized environments for specific members or distinct phases [4,71]. Semi‐permeable membranes can be used to physically segregate different members of a microbial consortium while allow interspecies nutrients exchange and chemical communication [72]. For instance, a heterogeneous bioreactor configured with a polydimethylsiloxane (PDMS) membrane could generate an oxygen gradient, which fulfilled the oxygen requirements of a microbial consortium composed of an aerobic fungus, a facultative anaerobic lactic acid bacterium and an obligate anaerobic bacterium [73]. Hydrogel encapsulation is also a potential technology to satisfy the different demands for oxygen in light‐driven microbial consortia, due to the relatively anoxic environment inside the hydrogel. Recently, we developed a conductive hydrogel to encapsulate *E. coli*, *S. oneidensis* and *G. sulfurreducens* for preventing them to be exposed to oxygen, which enables these heterotrophic bacteria to be co‐cultured with cyanobacteria simultaneously for a long‐term photoelectrical conversion [64]. Furthermore, a hierarchical structure could be created using multi‐layered hydrogel [74], thus the organization of species from inside to outside can be arranged as anaerobic, facultative and aerobic bacteria, respectively. Moreover, the assembly and large‐scale fabrication of hydrogel‐based microbial consortia can be accomplished using 3D printing technology. In device level, microfluidic systems, in the form of connected microwells or droplets, provide a promising platform to more accurately control the distribution and interaction of species in small scale [75]. It might be practical to organize separated bioreactors containing different microorganisms in series or as multi‐stage continuous cultures in large‐scale industrial processes [4]. Besides abiotic approaches, biotic solutions are also need to be explored. Several genetic engineering approaches have been developed in *E. coli* to manipulate oxygen utilization. This includes inactivating terminal oxidases and adjusting the intracellular concentration of ubiquinone, thus blocking or weakening the aerobic respiration of the corresponding engineered strains [76]. For a strict anaerobe, respiratory protection can be provided by introducing a partner strain with a high oxygen consumption rate but a low byproduct formation rate [77]. In addition, developing metabolic models with oxygen as a core parameter is important to quantitatively predict and control the growth rates and the community structure, and eventually improving the performance of light‐driven synthetic microbial consortia.

## CONCLUSIONS

Light‐driven synthetic microbial consortia have shown great potentials especially in the applications of wastewater treatment, chemical production from CO_2_, biohydrogen production and bioelectricity generation. An intrinsic characteristic of light‐driven synthetic microbial consortia is the oxygen production by photosynthetic partners, thereby creating an aerobic environment. Such an aerobic environment exhibits the distinct effects on the performance of various types of light‐driven microbial consortia, *i.e*., showing the positive effects on wastewater treatment and aerobic chemical production, while showing the negative effects on biohydrogen production, bioelectricity generation and anaerobic chemical production. To address the oxygen dilemma, we proposed several biotic/abiotic approaches, such as membrane segregation, hydrogel encapsulation, microfluidic compartmentalization and genetic manipulation of respiration chain. We trust well‐addressed oxygen dilemma will enable us to develop more efficient light‐driven synthetic microbial consortia and achieve various biotechnological applications, especially for bio‐solar cells and cost‐effective anaerobic chemical production from CO_2_.

## COMPLIANCE WITH ETHICS GUIDELINES

The authors Huawei Zhu and Yin Li declare that they have no conflict of interests.

This article is a review article and does not contain any studies with human or animal subjects performed by any of the authors.
